# Tortoise-Inspired Magnetically Actuated Soft Robot Enabled by Segmented Legs with Programmable Bending

**DOI:** 10.3390/biomimetics11090608

**Published:** 2026-08-27

**Authors:** Nanhao Zhou, Han Huang

**Affiliations:** 1 School of Integrated Circuits, Nanjing University of Information Science and Technology, Nanjing 210055, China; 2 State Key Laboratory of Precision Manufacturing for Extreme Service Performance, Central South University, Changsha 410083, China

**Keywords:** soft robotics, magnetically actuated soft robot, tortoise-inspired robot, multi-environment locomotion

## Abstract

Inspired by the natural curvature and jointed configuration of tortoise limbs, this paper presents a magnetically controlled soft robot based on a “programmable intrinsic curvature” design paradigm. When corrugated flexural notches are introduced into a single-material elastomeric leg, localized stress concentration enables each leg to acquire a predefined curved shape during fabrication. Combined with corrugated-straw-based sacrificial molding and magnetic-field-assisted curing, the strategy allows independent tuning of bending angle and leg length. Four legs are radially integrated onto a soft torso. Experimental results show that the stepping–pushing asymmetry of a single leg is governed by the predefined curvature, with the snap-through state corresponding to the equilibrium between magnetic attraction and paramagnetic deflection. The robot achieves speeds of 1.1 mm/s on dry ground, 9.5 mm/s in semi-submerged water, and 56 mm/s in fully submerged water (at 0.8 Hz, 340 mT), a ~50-fold increase in swimming speed compared with its terrestrial locomotion speed. It also demonstrates sharp turning, payload transport, rough surface traversal, and self-righting. This work elevates intrinsic curvature from passive geometry to an active design variable for soft robots in complex multi-environment scenarios.

## 1. Introduction

Soft robotics has emerged as a transformative paradigm in recent years, offering significant advantages over traditional rigid robots in terms of compliance, safety in human–robot interaction, and adaptability to unstructured environments [1,2]. Among various actuation strategies, magnetic actuation has garnered particular attention due to its wireless operation, rapid response, deep tissue penetration, and precise spatiotemporal control [3,4,5]. Magnetically actuated soft robots have been demonstrated in a range of applications, including targeted drug delivery [6,7], minimally invasive surgery [8,9], and cargo manipulation in confined spaces [10,11]. Despite these advances, most existing magnetic soft robots are designed and optimized for a single operational medium—dry ground, shallow water, or fully submerged conditions—and their locomotion performance degrades significantly when transitioning between media. Achieving effective and switchable locomotion across multiple environmental media remains a substantial challenge for the field [11,12].

Nature offers abundant solutions to multi-environment locomotion. Tortoises, for instance, are capable of moving efficiently on both land and in water, relying on the synergy between the natural curvature of their limbs and their jointed configuration. The curved limb geometry provides structural support during terrestrial walking and generates propulsive force during aquatic paddling, while the articulated joints enable the large-amplitude limb swings required for both gaits [13]. This biomechanical strategy suggests that embedding a predefined curvature into soft robotic limbs could confer similar adaptability across different media. In recent years, extensive efforts have been devoted to multimodal locomotion of magnetically controlled soft robots. Inspired by the green sea turtle, Guo et al. [14] developed a six-legged soft robot with six magnetized feet, achieving stable locomotion on dry ground, in semi-submerged conditions, and under fully submerged conditions within a magnetic field intensity range of 1.84–6.44 mT. The robot could climb slopes up to 30° and traverse U-shaped bends, reaching speeds of approximately 25 mm/s (10 Hz). However, this design relies on six independent magnetized feet, resulting in a relatively complex structure, and the bending deformation of the legs mainly depends on the overall compliance of the structure rather than a predefined geometric configuration. Inspired by amphibious organisms such as the mudskipper, researchers [15] developed a finger-sized magnetically controlled soft robot based on soft slime materials and investigated its amphibious locomotion strategies. Nevertheless, the mechanical properties of the slime material limit its payload capacity and motion precision. Inspired by fin-wave propulsion, Wang et al. [16] designed a small-scale soft robot capable of adaptive amphibious locomotion on liquid and solid surfaces under a single rotating magnetic field, enabling movement across water, oil, and dry surfaces. However, the locomotion mode of that robot is rather limited, relying primarily on undulatory fin-based propulsion, which makes it difficult to achieve gait switching or complex terrain adaptation as seen in legged animals. Inspired by millipedes, Liu et al. [17] adopted a multi-legged structure with a flexible torso, with feet made of magnetic soft materials, generating travelling waves under a rotating magnetic field for efficient locomotion in complex rugged environments. Yet, the multi-legged configuration introduces fabrication complexity and coordination challenges in motion control. In addition, researchers combined bistable kirigami structures with hard-magnetic actuation to achieve programmable multimodal locomotion, including amphibious behavior and L-shaped trajectory tracking [18]. The mode switching in that approach relies on rapid transitions between bistable states, which restricts deformation to predefined stable configurations and does not allow continuously tunable bending. In the context of tortoise-inspired robots, Xu et al. developed a small-scale magnetically soft robotic turtle with programmable magnetization, using an Ecoflex body and four VHB magnetized limbs, capable of straight swimming, turning swimming, and carrying loads exceeding twice its own weight [19]. However, the limb bending in that design also depends on the overall magnetization programming of the material rather than the geometric design of the structure itself, lacking active control over the intrinsic curvature of the legs.

In most of the above studies, the deformation and locomotion of magnetically controlled soft robots rely on global programming of the magnetization distribution or real-time tuning of the external magnetic field [20,21,22], while the predefined geometric curvature of the structure itself is rarely exploited as an independent design variable [23,24]. Correspondingly, in legged soft robots, bending deformation is typically induced actively by the external magnetic field, rather than being imparted as an intrinsic curved shape during fabrication. This implies that switching locomotion modes across different media often requires changing the magnetic actuation strategy or relying on passive compliance of the structure, rather than having the leg’s own predefined geometry guide the direction and amplitude of deformation [25,26,27]. Therefore, how to achieve programmability of intrinsic curvature in legged soft robots through structural design itself, rather than solely through material programming or magnetic field tuning, and on this basis construct bioinspired robots with multi-medium adaptability, remains an open challenge [1,7,28].

Here, we introduce a design paradigm of “programmable intrinsic curvature” for magnetically controlled soft robotic legs. By incorporating corrugated flexural notches into a single-material elastomeric leg, we generate localized stress concentration that enables each leg to acquire a predefined curved shape during fabrication. Combined with a corrugated-straw-based sacrificial molding method and magnetic-field-assisted curing, our strategy allows independent tuning of both the bending angle and leg length, while embedded magnetic particles provide programmable responsiveness to external magnetic fields. Four such legs are radially integrated onto a soft torso, forming a fully soft bioinspired tortoise robot. We systematically characterize the bending behavior of individual legs, revealing that the asymmetry between stepping and pushing is governed by the predefined intrinsic curvature, with the snap-through state corresponding to the equilibrium between magnetic attraction and paramagnetic deflection. We then demonstrate that the integrated robot achieves effective locomotion across three distinct media: dry ground, semi-submerged water, and fully submerged water, with speeds reaching 1.1 mm/s, 9.5 mm/s, and 56 mm/s, respectively, representing an approximately 50-fold increase in swimming speed compared with its terrestrial locomotion speed. The robot also exhibits sharp turning, payload transport, rough-surface traversal, and self-righting after overturning. By elevating intrinsic curvature from a passive geometric feature to an active design variable, this work provides a viable pathway for soft robots intended for complex multi-environment scenarios, with potential applications in environmental monitoring, search-and-rescue, and biomedical interventions.

## 2. Design, Fabrication, and Characterization of the Bioinspired Soft Leg

**Figure 1:**
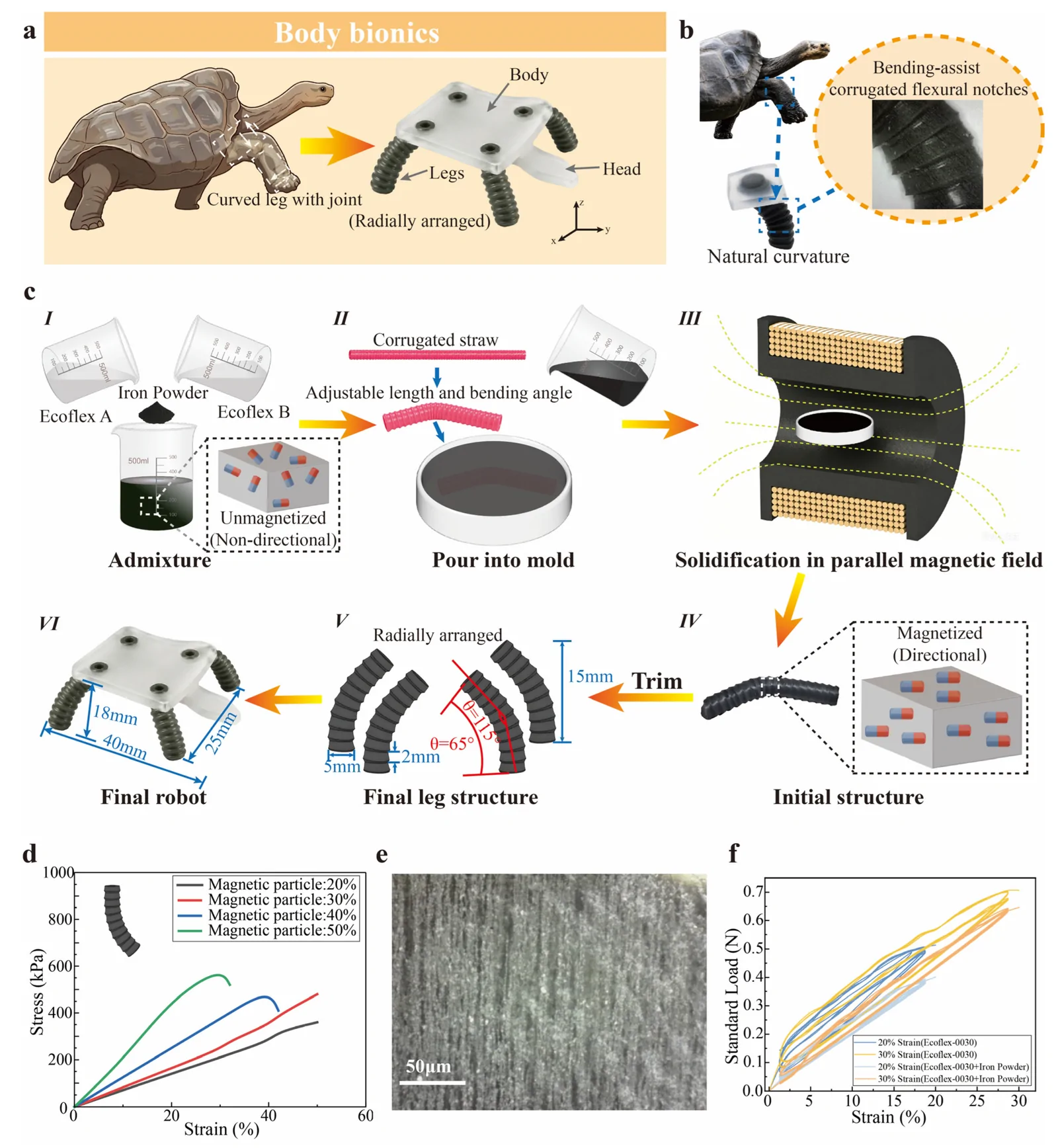
Tortoise-inspired design and mechanical characterization of the magnetic soft leg. (**a**) Tortoise-inspired design of the soft robot. (**b**) Flexible leg structure designed based on the intrinsic bending angle of tortoise limbs during standing. (**c**) Fabrication process of the Tortoise-Inspired Soft Robot. (**d**) Mechanical characterization of Ecoflex composites with different magnetic particle contents. (**e**) SEM image of the magnetic composite after magnetic-field-induced activation. (**f**) Cyclic tensile tests demonstrating the recoverable deformation capability of the composite.

The locomotion mechanics of tortoise limbs rely heavily on the synergy between the natural curvature and the jointed configuration of their legs: the curved morphology provides static support and reduces impact energy dissipation during the gait cycle through passive compliance. Inspired by this, we propose a bioinspired magnetically controlled soft leg based on corrugated flexural notches, arranged radially around a miniature torso to reproduce the spatial layout and motion redundancy of tortoise limbs (Figure 1a). Specifically, by combining morphological biomimicry with material programming, the soft leg simultaneously achieves a predefined intrinsic curved configuration and magnetically responsive controllable bending capability (Figure 1b). Its intrinsic bending does not rely on conventional hinges or revolute joints, but is realized through circumferentially distributed corrugated notches along the leg axis. The notches locally reduce the sectional modulus, generating a stress concentration effect that induces controllable bending strain energy at the macroscale, endowing the leg with a predefined curved shape prior to magnetic stimulation. This structure–compliance synergy enables bending deformation to distribute continuously along the leg length rather than being localized at discrete joint nodes, significantly enhancing the conformability of the soft actuator on complex terrains.

Figure 1c illustrates the integrated “mold constraint–magnetic field programming” fabrication method for the magnetic soft leg. Ecoflex 00-30 and nano-iron particles at varying mass fractions (20 wt–50 wt%) are mechanically stirred under vacuum to achieve homogeneous dispersion. At this stage, the suspended magnetic particles, influenced by the competing effects of Brownian motion and gravitational sedimentation, exhibit randomly oriented easy axes (randomly oriented), manifesting macroscopically as superparamagnetism. A commercially available corrugated drinking straw (corrugated straw), cut and preset to a constant planar curvature, is immersed into the mixture as a sacrificial mold, providing a simple, low-cost, and readily accessible approach for programming the geometry of the magnetic soft legs. By adjusting the cutting length and preset curvature of the straw, the leg length and bending angle can be conveniently customized. The cutting length and preset curvature of the straw determine the adjustable length and bending angle of the final leg, while the corrugations serve as a negative mold for the surface micro-grooves. The entire mold system is then cured at room temperature under a high-intensity uniform parallel magnetic field, during which the magnetic particles realign along the field lines to form one-dimensional chain-like ordered structures, encoding and fixing the anisotropy of the magnetic field direction into the elastomeric matrix. After demolding, a flexible magnetic soft leg is obtained, featuring a natural curved morphology, surface corrugated notches, and intrinsic magnetic anisotropy. Finally, four legs are mounted onto a pure Ecoflex torso in an orthogonal radial arrangement, complemented by a lightweight head counterweight, which also serves as a passive stability pivot by providing ground contact support during overturning and offering geometric constraints for subsequent self-righting mechanisms. Based on the experiments presented in Supplementary Figures S1–S3, the final robot adopts a leg length of 1.8 mm, a leg spacing of 2.5 mm, and an intrinsic bending angle of 60°. This set of parameters achieves a favorable balance among locomotion efficiency, structural stability, and deformation capability. The overall dimensions of the robot are 40 mm × 25 mm × 18 mm.

Figure 1d examines the effect of nano-iron particle content on the mechanical properties of the Ecoflex composite. As the content increases from 20 wt% to 50 wt%, the elastic modulus rises while the fracture strain decreases. The 20 wt% and 30 wt% specimens did not fracture within the testing range, while the 40 wt% and 50 wt% specimens exhibited fracture strains of 39% and 28%, respectively. Considering the soft leg’s requirements for low bending stiffness and large deformation capacity, we selected the 20 wt% formulation to prioritize compliance while maintaining sufficient magnetic responsiveness. SEM images (Figure 1e) reveal that after curing under a parallel magnetic field, the nano-iron particles are uniformly distributed within the Ecoflex matrix with notable alignment along the field direction, effectively avoiding stress concentration caused by particle agglomeration and verifying the efficacy of the magnetic-field-assisted curing process in microstructural regulation. Cyclic loading–unloading tests (Figure 1f) demonstrate that after 100 cycles, the hysteresis loops nearly overlap for all formulations, with minimal residual strain, confirming that the viscoelastic recovery capability of the Ecoflex matrix is not significantly degraded by the incorporation of nano-iron particles, thereby ensuring repeatable deformation of the bioinspired leg under alternating magnetic fields.

## 3. Magnetically Actuated Bending Behavior and Kinematics of a Single Leg

**Figure 2:**
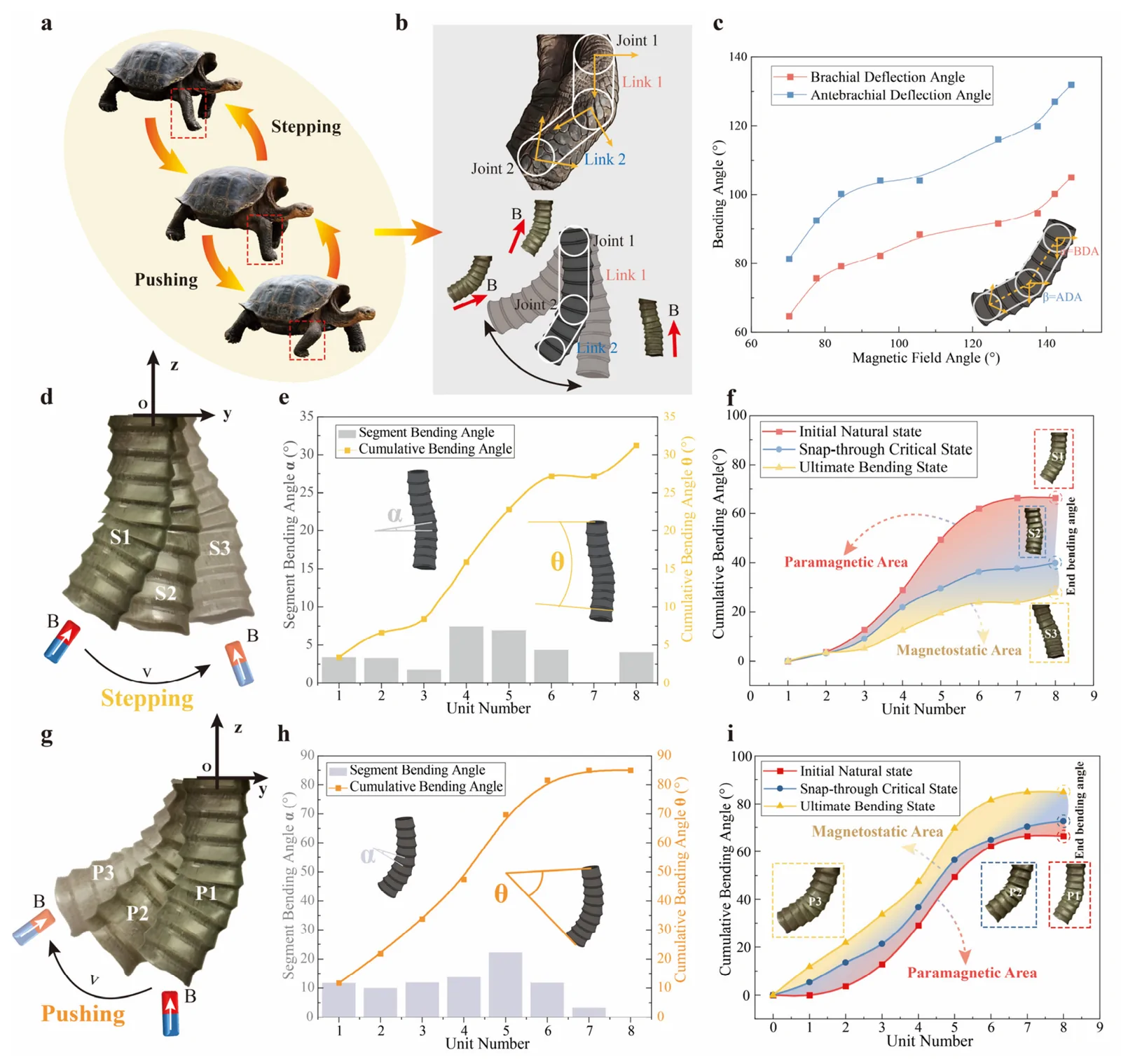
Magnetically induced bending behavior and kinematic analysis of a single leg. (**a**) Tortoise-inspired locomotion modes during stepping and pushing. (**b**) Kinematic model of the leg based on a two-link and two-joint system. (**c**) Magnetic-field-dependent bending angles of brachial and antebrachial segments. (**d**) Bending configuration during stepping motion. (**e**) Segmental bending distribution at the maximum extension state during stepping. (**f**) Magnetic-field-induced bending transition during stepping motion. (**g**) Bending configuration during pushing motion. (**h**) Segmental bending distribution at the maximum bending state during pushing. (**i**) Magnetic-field-induced bending transition during pushing motion.

Tortoise gaits consist of two alternating phases: stepping and pushing (Figure 2a). In this study, we simplify the tortoise leg as a two-link, two-joint system: the torso–leg connection as Joint 1, the knee as Joint 2, and the thigh and shank as Link 1 and Link 2, respectively (Figure 2b). This kinematic model is equally applicable to the magnetic soft leg: the apex of its natural curvature corresponds to the knee, and the two leg segments on either side correspond to the thigh and shank. By applying a rotating magnetic field, the soft leg can bend forward or backward, replicating stepping and pushing, respectively. To quantitatively characterize the bending behavior, we define the brachial deflection angle (BDA)—the angle between Link 2 (shank) and the horizontal—and the antebrachial deflection angle (ADA)—the angle between Link 1 (thigh) and the horizontal (Figure 2c). All BDA/ADA measurements are conducted with the soft leg hanging downward, matching its actual working posture during locomotion. By quantifying how the orientation of the rotating magnetic field governs the angular response of the two leg segments, Figure 2c verifies the paramagnetic responsiveness of the soft leg and establishes the foundation for analyzing the two distinct bending modes—stepping and pushing—in the subsequent panels. As the magnetic field angle increases from 70° to 150°, the BDA increases monotonically from 63° to 105°, while the ADA increases from 81° to 130°. The ADA remains consistently larger than the BDA throughout the entire range, a difference attributed to the geometric asymmetry inherent in the intrinsic curved configuration. Both curves exhibit a slower variation in the central interval (90–120°) and faster changes at the two ends. This “slow-in-the-middle, fast-at-the-ends” response is directly related to the intrinsic bending angle (115°): when the field approaches the intrinsic configuration, the corrugated flexural notches provide compliant buffering that cushions the angular response; when the field deviates further from the intrinsic shape, the bending deformation must overcome greater structural stiffness, resulting in more pronounced angular changes. The high similarity between the BDA and ADA curves indicates uniform magnetic field distribution and elastic modulus within the material, validating the structural consistency achieved by the curing process.

Consistent with the configuration adopted in Figure 2c, the stepping and pushing tests are performed with the soft leg mounted in the same downward-hanging orientation. The stepping process sequentially passes through three characteristic states: the initial natural state S1, the snap-through critical state S2, and the ultimate bending state S3 (Figure 2d). S2 corresponds to the mechanical equilibrium point between magnetic attraction and paramagnetic deflection, characterized by a sudden drop in structural stiffness and rapid configuration transition; at S3, the leg is nearly straight. To quantify local deformation, each segment divided by the corrugated notches is treated as a compliant unit, and the relative rotation angle between adjacent segments is defined as the segment bending angle; the cumulative sum of these angles yields the cumulative deflection angle (Figure 2e). During stepping, the cumulative deflection angle increases from 25° at S1 to 70° at S3 (Figure 2f), without reverse bending. The snap-through at S2 is not triggered by a fixed cumulative deflection threshold, but rather by the global equilibrium between magnetic attraction and paramagnetic deflection, and its position shifts with varying magnetic field strength and material parameters. The pushing process follows the same analytical framework (Figure 2g–i); however, due to the geometric asymmetry of the intrinsic curvature, the bending direction is opposite and the amplitude is significantly smaller: the cumulative deflection angle increases from 25° at S1 to 80° at S3 (range 63–80°), considerably narrower than the 25–70° range of stepping. This difference arises because the intrinsic curved configuration provides a larger deformation space for forward bending (stepping direction), while imposing a geometric constraint on reverse bending (pushing direction)—stepping aligns with the intrinsic curvature, amplifying the amplitude; pushing opposes it, requiring the leg to overcome the restoring moment, thus limiting the amplitude. Consequently, the asymmetry between stepping and pushing is determined by the preset intrinsic curvature rather than by material or magnetic field inhomogeneities. In summary, the bending direction and amplitude of the soft leg vary monotonically with the orientation of the rotating magnetic field, and the leg naturally returns to its intrinsic configuration upon removal of the field. When the field varies periodically, the soft leg undergoes reciprocating bending that drives the overall robot in a walking gait—this periodic actuation mechanism will be analyzed in the next section in conjunction with the overall robot locomotion.

## 4. Terrestrial Gait Locomotion

**Figure 3:**
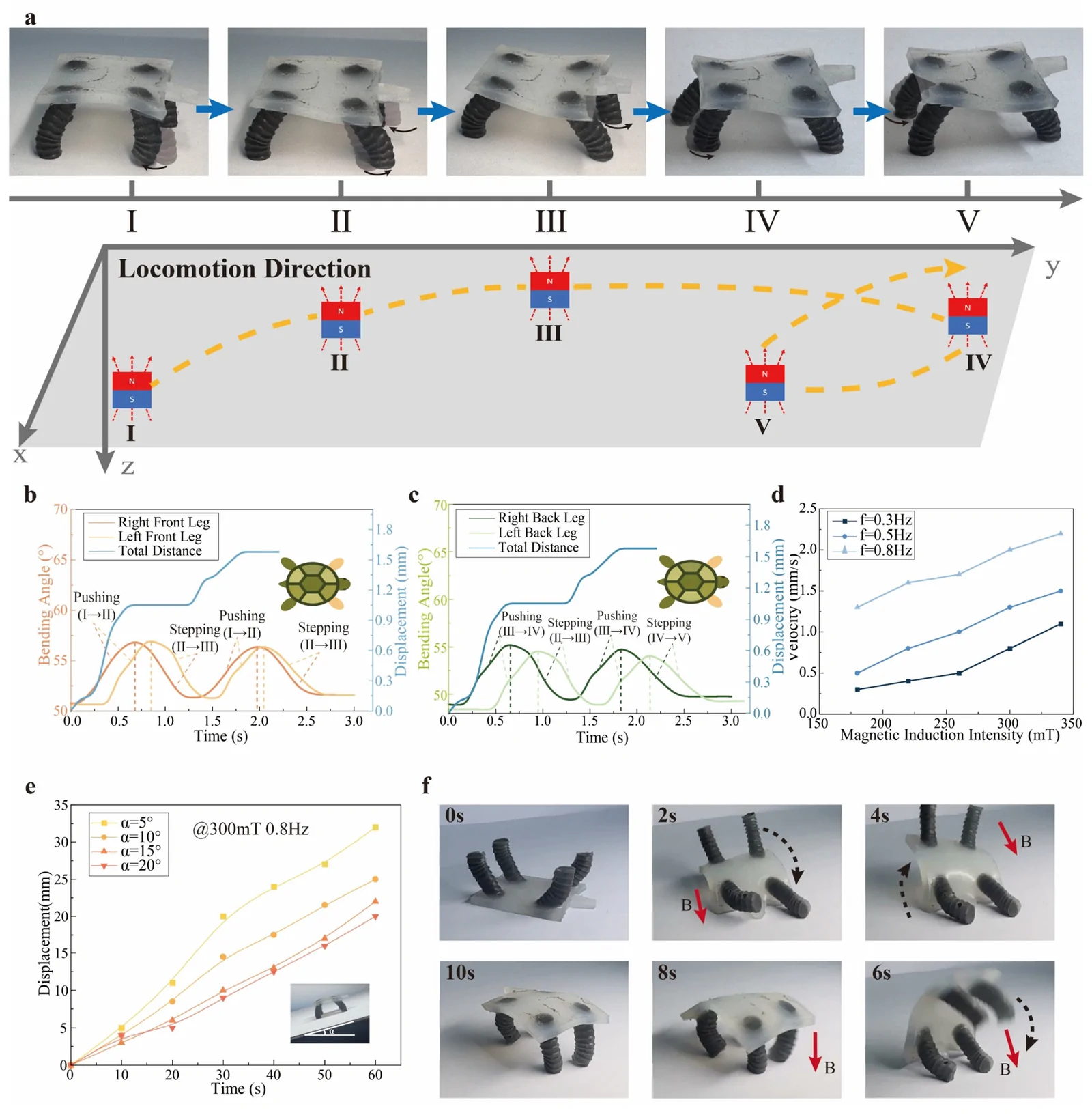
(**a**) Experimental demonstration and magnetic-field trajectory for robot locomotion. (**b**) Synchronous analysis of foreleg bending and displacement during locomotion. (**c**) Synchronous analysis of hindleg bending and displacement during locomotion. (**d**) Effect of magnetic field intensity and frequency on locomotion velocity. (**e**) Locomotion performance under different inclined surfaces. (**f**) Magnetically controlled self-righting after full flexible body contact.

Based on the bending behavior of a single magnetically actuated soft leg under a rotating magnetic field, we further analyzed the locomotion performance of the overall robot under magnetic actuation. In one complete motion cycle, the four legs sequentially execute pushing and stepping actions (Figure 3a). The magnet undergoes a superposition of circular motion in the horizontal plane and unidirectional linear motion, where the circular motion provides the alternating swing timing of the legs, and the unidirectional linear motion continuously shifts the magnet forward relative to the robot, thereby driving the entire robot forward. The positions of the magnet relative to the robot are sequentially: right-front (I), straight-front (II), left-front (III), right-rear (IV), and left-rear (V). After completing a full cycle, the magnet advances by one step distance, corresponding to the robot’s net displacement. Under this timing sequence, the right-front leg first pushes, the left-front leg then steps, followed by the left-front leg pushing, and the rear legs stepping in sequence, forming a stable gait rhythm. The periodic motion of the magnet imparts fixed phase differences to the legs, with the front legs lagging by approximately 0.25 s and the rear legs by approximately 0.3 s.

The coordinated motion of the two front legs was quantified by synchronously recording the cumulative deflection angle and displacement (Figure 3b). Under a 0.8 Hz magnetic field, the cumulative deflection angles of both front legs increased from 50° to 57° and then returned to 50°, with the two processes nearly overlapping completely, indicating good symmetry in front-leg motion. During the pushing phase, the cumulative deflection angle increased and the robot moved forward; during the stepping phase, the angle decreased and the robot remained stationary while the legs adjusted their posture in preparation for the next push. Due to the periodic rotation of the magnet, the two front legs moved alternately, with the right front leg leading the left. The travel distance per cycle was approximately 1 mm. The two rear legs exhibited similar behavior (Figure 3c), with cumulative deflection angles ranging from 49° to 55°, slightly smaller in amplitude than the front legs, attributed to the relatively lower magnetic field intensity at the rear legs. The coordinated work of the front and rear legs demonstrates that the robot’s motion is driven collectively by all four legs.

Magnetic field parameters have a significant regulatory effect on locomotion speed (Figure 3d). At three frequencies of 0.3 Hz, 0.5 Hz, and 0.8 Hz, the locomotion speed increased with magnetic field intensity (from 180 mT to 340 mT), reaching 1.1 mm/s at 0.8 Hz and 340 mT, and 0.25 mm/s at 0.3 Hz and 180 mT. Under the condition of 300 mT and 0.8 Hz, the robot moved upward on inclined planes from 5° to 20° (Figure 3e), with the displacement within 60 s decreasing from 32 mm to 20 mm, indicating that increasing inclination reduces the effective driving force; nevertheless, the robot maintained continuous climbing even on a 20° slope. Self-righting capability was verified through overturning recovery experiments (Figure 3f): the robot overturned at 2 s, with the flexible torso touching the ground and the legs in an irregularly splayed state. A translational magnetic field was then applied to drive the two legs on one side. Under magnetic attraction, these legs bent toward the direction of the applied magnetic field, generating a net bending moment that shifted the robot’s center of gravity relative to the supporting edge. The robot subsequently rotated about the supporting edge under the continuously translated magnetic field and recovered to its upright state at 8 s. The unilateral actuation of the two legs on one side is critical for generating the net recovery torque, while the orthogonal radial arrangement of the four legs is maintained throughout the recovery process. This mechanism demonstrates the robustness of the present design in unstructured environments.

## 5. Terrestrial–Aquatic and Comprehensive Locomotion

**Figure 4:**
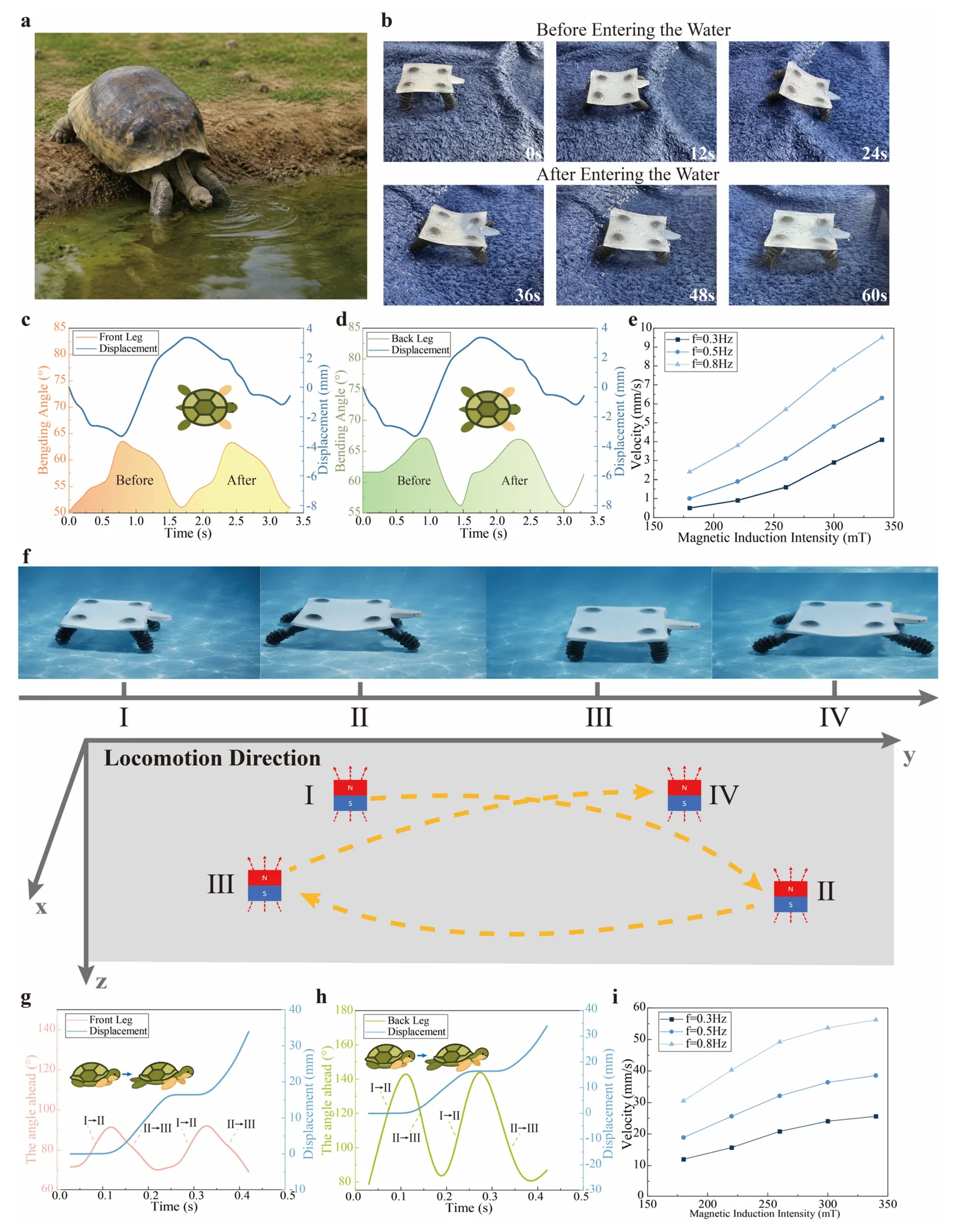
Adaptive terrestrial-aquatic locomotion of the magnetically controlled soft robot. (**a**) Tortoise-inspired amphibious locomotion across terrestrial and aquatic environments. (**b**) Terrestrial-to-aquatic transition with partial body immersion. (**c**) Foreleg bending behavior and displacement during partial immersion. (**d**) Hindleg bending behavior and displacement during partial immersion. (**e**) Locomotion velocity under different magnetic conditions during partial immersion. (**f**) Swimming sequence and programmed magnetic-field trajectory in fully submerged water. (**g**) Foreleg paddling behavior during underwater swimming. (**h**) Hindleg paddling behavior during underwater swimming. (**i**) Swimming velocity under different magnetic conditions.

Based on the locomotion on dry ground, we further investigated the robot’s locomotion capability when partially submerged in shallow water and when fully submerged in water (Figure 4a,b). Under different submersion depths, the fluid environment exerts a significant influence on the dynamic behavior of the legs, and the locomotion mode undergoes adaptive changes accordingly.

When the robot is in a partially submerged state, the buoyancy of water partially counteracts gravity, significantly reducing the normal pressure between the legs and the ground, thereby lowering frictional resistance. This results in a notable decrease in the minimum magnetic field intensity required for actuation compared with the dry-ground condition, while both the front and rear legs maintain coordinated motion without significant phase lag (Figure 4c,d). The introduction of buoyancy not only mitigates the constraints of ground friction on actuation efficiency but also enables the legs to more easily overcome static friction at the contact surface during the reset phase, thereby reducing energy loss during the stepping–pushing transition. Consequently, under identical input magnetic field conditions, the locomotion speed in the semi-submerged condition is significantly higher than that on dry ground. At three magnetic field frequencies of 0.3 Hz, 0.5 Hz, and 0.8 Hz, the locomotion speed increased with magnetic field intensity (from 180 mT to 340 mT), with the speed at 0.3 Hz increasing from 0.5 mm/s to 4 mm/s, and at 0.8 Hz from 2.4 mm/s to 9.5 mm/s (Figure 4e).

When the robot is fully submerged in water, as the composite material density measured by the Archimedes drainage method is approximately 1.15 g/cm^3^, slightly higher than water, the robot sinks to the bottom but experiences extremely low normal pressure, making frictional resistance negligible. At this point, the locomotion mode transitions from the stepping–pushing gait to a turtle-like paddling posture (Figure 4f). The magnet undergoes a superposition of a complete elliptical motion in the yoz plane and a linear motion along the y-direction. The front endpoint (II) and rear endpoint (III) of the elliptical trajectory correspond to the extended states of the legs: from I to II, the legs extend outward while the robot torso remains stationary; from II to III, the legs paddle inward via elastic restoring force—this is the only propulsive phase in the entire cycle, driving the robot forward; from III to IV, the legs retract and reset, also generating no displacement. The time allocation for I→II, II→III, and III→IV in the entire cycle is approximately 0.06 s, 0.09 s, and 0.06 s, respectively. During this process, the front legs paddle backward to generate propulsive force, while the rear legs extend forward to maintain postural balance, and the two work in coordination to form an efficient propulsion mode. The cumulative deflection angle of the front legs increases from 72° to 94°, with a variation amplitude of 22°; that of the rear legs increases from 80° to 140°, with a variation amplitude of 60° (Figure 4g,h), reflecting that the rear legs undertake a larger swing stroke during the paddling process. The swimming speed is significantly improved under the same magnetic field conditions compared with the terrestrial and semi-submerged conditions (Figure 4i): at 0.3 Hz and 180 mT, the speed is approximately 12 mm/s, and at 0.8 Hz and 340 mT, it reaches 56 mm/s, which is mainly attributed to the low resistance of the aqueous environment and the high propulsion efficiency of the paddling mode.

Integrating the locomotion performance under the three conditions of dry ground, semi-submersion, and full submersion, the locomotion mode of this bioinspired robot undergoes significant adjustments with changes in the environmental medium: on dry ground, it walks with a stepping–pushing gait, with speed limited by ground friction and static friction loss during leg reset; under semi-submerged conditions, buoyancy effectively reduces normal pressure and friction loss, leading to improved speed under the same magnetic field input; when fully submerged, the locomotion mode transitions to paddling propulsion, where the low-resistance aquatic environment and the high-frequency paddling mechanism driven by elastic recovery work together to achieve the maximum speed among the three conditions. The above results demonstrate that this flexible magnetic soft robot possesses the capability to switch locomotion modes in multi-environmental media, providing an experimental foundation for its potential applications in complex underwater environments.

**Figure 5:**
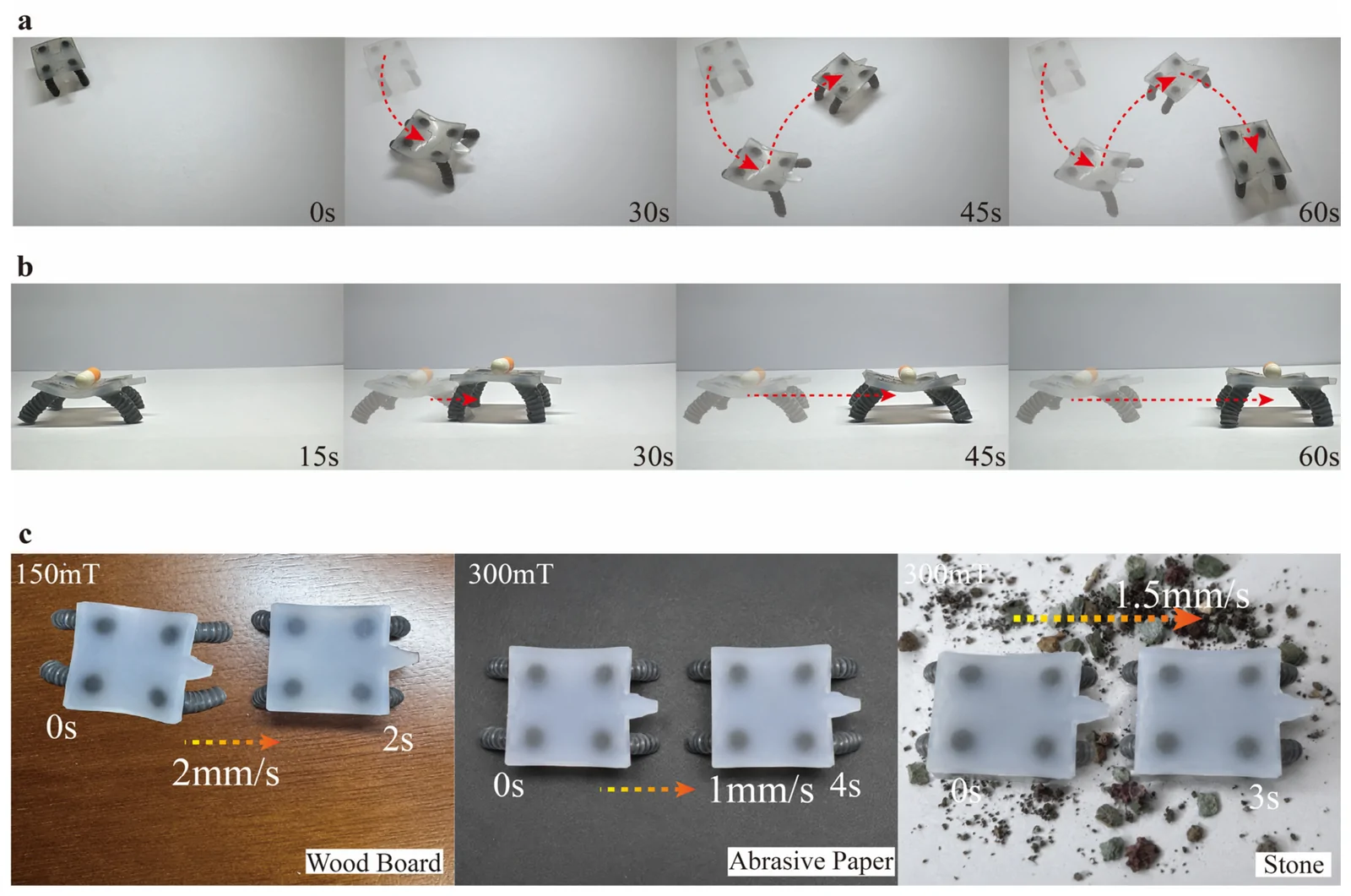
Multifunctional locomotion capability of the magnetically controlled soft robot. (**a**) Magnetically controlled turning maneuver through asymmetric actuation. (**b**) Locomotion with cargo transportation capability. (**c**) Locomotion performance on different substrate surfaces.

In addition to straight-line locomotion and multi-environmental locomotion, this robot also possesses comprehensive locomotion capabilities including turning, payload transportation, and traversal over rough terrains. Turning is achieved by deflecting the rotating magnetic field toward one side: when the magnetic field direction is asymmetrically biased toward one side of the robot, the legs on that side obtain greater magnetic driving force, and the friction with the ground is correspondingly enhanced, while the driving force on the non-deflected side is relatively weakened; the friction difference between the two sides generates a yaw moment that drives the robot to complete the turn. As shown in Figure 5a, this turning strategy enables two sharp-angle turns to be completed within 30 s, without posture instability or overturning during the turning process, reflecting the static stability provided by the flexible torso and multi-legged support. The payload capability was verified by crawling while carrying a pill (Figure 5b): under load, the leg motion rhythm remains essentially consistent with that under no-load conditions, and the pill maintains stable transport throughout the motion, indicating that vibrations and tilting generated by the gait are effectively buffered by the flexible torso and do not disturb the carried payload. In locomotion tests on different substrates (Figure 5c), the robot’s forward speeds on a wooden board, sandpaper, and a pebble-covered surface were 2 mm/s, 1 mm/s, and 1.5 mm/s, respectively, demonstrating that even on rough or loose substrates, the local compliance provided by the corrugated flexural notches enables the legs to maintain effective contact with the surface, avoiding slipping or sinking, and thereby ensuring basic locomotion capability.

## 6. Conclusions

This study introduces and validates a design paradigm of “programmable intrinsic curvature” for magnetically controlled soft robotic legs. A key innovation is a low-cost sacrificial molding strategy using commercially available corrugated drinking straws, which enables convenient and controllable customization of the leg bending angle and length. By incorporating corrugated flexural notches into a single-material elastomer, localized stress concentration is generated, enabling each leg to acquire a predefined curved configuration during fabrication. Combined with magnetic-field-assisted curing, this strategy allows independent tuning of the intrinsic bending angle and leg length. Single-leg experiments reveal that the asymmetry between stepping and pushing is governed by the predefined geometry rather than by material or magnetic field inhomogeneities, and the snap-through critical state corresponds to the global mechanical equilibrium between magnetic attraction and paramagnetic deflection.

At the system level, four flexible legs integrated in an orthogonal radial arrangement enable effective locomotion across three distinct media: dry ground, semi-submerged water, and fully submerged water, with speeds reaching 1.1 mm/s, 9.5 mm/s, and 56 mm/s, respectively (at 0.8 Hz, 340 mT). Swimming speed represents a roughly 50-fold increase over terrestrial locomotion, highlighting the synergistic advantage of structural compliance and low-resistance aquatic environments. Moreover, the coordinated design of leg geometry, preset magnetic fields, and magnetization directions enables autonomous self-righting after overturning, with the robot recovering its upright configuration within 8 s without additional actuators or complex control. The robot also demonstrates sharp turning, payload transport, and rough-surface traversal.

Limitations in sensing and control accuracy remain to be addressed; the current experimental platform does not yet support computer-programmed target trajectory planning and real-time feedback control for the magnetic actuator. Future work will upgrade the experimental infrastructure, integrate flexible sensing modules, and implement closed-loop trajectory tracking control for the magnetic actuator, while also exploring multi-robot coordination strategies. Overall, this work combines low-cost programmable structural fabrication with magnetically programmed autonomous recovery, offering a viable technological pathway for bioinspired soft robots operating across complex multi-environment scenarios.

## 7. Method

### 7.1. Fabrication of the Magnetic Robot

The magnetically programmed soft legs were fabricated through a mold-assisted shaping and magnetic programming process. Ecoflex 00-30 and iron nanoparticles were mixed at a 4:1 mass ratio and vacuum-stirred to achieve uniform dispersion. A commercial corrugated straw with predefined single-arc curvature was trimmed and fixed inside a container as a sacrificial mold, into which the magnetic composite precursor was poured. The straw’s periodic ring-shaped structures replicated the surface corrugations of the soft legs, while its predefined curvature determined the initial bending configuration. After complete infiltration, the mold-composite system was cured at room temperature under a uniform parallel magnetic field, which aligned the embedded magnetic particles along the field direction to encode the desired magnetization orientation. Following curing, the straw mold was peeled away, and the obtained magnetic elastomer was trimmed into leg components with identical lengths and designed bending angles. The trimmed leg units were inserted into a pre-prepared Ecoflex solution, fixed using a supporting structure, and subjected to secondary curing. Four magnetically responsive legs were then assembled onto a pure PDMS body in an orthogonal radial arrangement, with a lightweight head structure attached for passive stability. The final robot has overall dimensions of approximately 40 mm × 25 mm × 18 mm.

### 7.2. The Principle of Robot Structure

The overall dimensions and geometric parameters were determined based on biomimetic locomotion requirements, passive stability, and amphibious environmental adaptability. The intrinsic bending angle of the magnetic legs was set to approximately 115°, inspired by the naturally obtuse joint configuration of turtle hind limbs during locomotion. This angle provides sufficient angular reserve in both bending directions to achieve a relatively symmetric motion range during periodic actuation—approximately 20–30° of angular variation during forward stepping and backward pushing—while avoiding excessive unidirectional deformation that could reduce propulsion efficiency. A lightweight head structure was introduced as a passive balancing element, inspired by the supporting mechanism of turtle head–limb coordination, providing an additional contact point under magnetic actuation to facilitate recovery after overturning and suppressing excessive body rotation in complex underwater environments. The leg height was designed to be approximately 15 mm, offering sufficient ground clearance for walking on uneven substrates while maintaining adequate workspace for generating propulsive paddling in fully submerged swimming. Overall, the structural dimensions were optimized through a balance between biomimetic morphology, magnetic deformation capability, and amphibious locomotion requirements, rather than by simple geometric scaling.

### 7.3. Magnetic Control Strategies for Dual Locomotion Modes

Two magnetic control strategies were developed for substrate-assisted walking and free swimming, respectively, by changing the rotation plane of the external magnetic field to actively regulate the deformation direction and periodic motion of the magnetic legs. In the walking mode (xy plane), the external magnetic field is periodically rotated within the horizontal plane. As the magnetization direction of each leg is vertically programmed, the rotating field induces periodic forward and backward bending, generating propulsion through cyclic leg–substrate contact and friction. Adjusting the rotation direction and frequency enables turtle-inspired terrestrial walking and underwater bottom crawling. In the swimming mode (yz plane), the rotation plane is switched to the vertical plane, inducing out-of-plane periodic oscillation of the legs. The cyclic extension and contraction generate asymmetric fluid interactions and forward thrust, with the fore and hind legs performing coordinated paddling motions similar to those of aquatic turtles. Both modes are achieved through wireless magnetic actuation without physical contact; simply changing the rotation plane allows the robot to switch between walking and swimming strategies.

## Data Availability

Data are contained within the article and Supplementary Materials.

## Supplementary Materials

The following supporting information can be downloaded at: https://www.mdpi.com/article/10.3390/biomimetics11090608/s1. Supplementary Movie S1; Supplementary Movie S2; S1: Magneto-Elastic Coupling Model of the Snap-Through Critical State; S2: Geometric Stiffness Model of Stepping–Pushing Asymmetry; S3: Hydrodynamic Analysis of Swimming Propulsion; S4: Torque Balance Analysis of Single-Sided Self-Righting; Figure S1: Fabricated soft robots with different structural designs; Figure S2: Design-space screening based on leg length and leg spacing; Figure S3: Effect of intrinsic bending angle on locomotion performance.
