# Supplementary material for: Tortoise-Inspired Magnetically Actuated Soft Robot Enabled by Segmented Legs with Programmable Bending
Source: Biomimetics (Basel). 2026 Aug 27;11(9):608. doi: 10.3390/biomimetics11090608 (PMC13604220; doi:10.3390/biomimetics11090608)
Supplement: Supplementary file 1 [file biomimetics-11-00608-s001.zip › Supplementary Materials.pdf]

Supplementary Material

S1. Magneto-Elastic Coupling Model of the Snap-Through Critical State

The single soft leg is simplified as a rigid pendulum supported by a torsional spring (equivalent torsional stiffness k_1), subjected to both magnetic torque τ_1 and elastic restoring torque τ_2 under an external magnetic field. Let θ denote the bending angle of the leg and θ_0 the intrinsic bending angle (115°). The elastic restoring torque can be approximated in linear form as:

$$\tau_1(\theta) = k_1(\theta - \theta_0) \qquad \text{(S1)}$$

The magnetic torque arises from the tendency of magnetized particles to align with the magnetic field direction and can be expressed as:

$$\tau_2(\theta) = m_1 B_1 \sin(\varphi - \theta) \qquad \text{(S2)}$$

where m_1 is the equivalent magnetic moment of the leg, B_1 is the applied magnetic field strength, and φ is the magnetic field direction angle. The equilibrium condition $\tau_1 = \tau_2$ gives:

$$m_1 B_1 \sin(\varphi - \theta) = k_1(\theta - \theta_0) \qquad \text{(S3)}$$

The number of real solutions to this equation varies with the magnetic field strength B_1. When B_1 is below the critical value B_2, the system has only one stable equilibrium solution near θ_0 (monostable). When B_1 reaches B_2, a new pair of stable and unstable equilibrium solutions appears on the opposite side (bistable), and the leg can jump from the original stable state to the new one under magnetic torque—this is the snap-through instability.

The critical condition is given by the extremum point of the equilibrium path:

$$\partial\tau_1/\partial\theta = \partial\tau_2/\partial\theta \qquad \text{(S4)}$$

Substituting Eqs. (S1) and (S2) yields:

$$m_1 B_1 \cos(\varphi - \theta) = k_1 \qquad \text{(S5)}$$

Solving Eqs. (S3) and (S5) simultaneously gives the critical magnetic field strength B_2 and the corresponding critical angle θ_1:

$$B_2 = k_1 / [m_1 \cos(\varphi - \theta_1)] \qquad \text{(S6)}$$

$$\tan(\varphi - \theta_1) = (\theta_1 - \theta_0) / [\cos(\varphi - \theta_1)] \qquad \text{(S7)}$$

Substituting the intrinsic bending angle $\theta_0 = 115°$, the experimental cumulative deflection angle range (25°–70°) and the magnetic field operating range (180–340 mT) into these equations yields the analytical relationship between the position of P2 and variations in B_1 and φ. The model predicts that when B_1 is low, P2 either does not exist or is located at a shallow bending configuration; as B_1 increases, P2 shifts toward larger bending angles, consistent with the experimental observation that "the position of P2 drifts with magnetic field strength."

Furthermore, the model predicts an important conclusion: the critical angle θ_1 corresponding to P2 is not fixed but increases monotonically with φ. This implies that during continuous scanning of the magnetic field direction, the specific configuration at which snap-through occurs depends not only on the field strength but also on the angle between the field direction and the intrinsic configuration—a prediction that awaits further experimental validation.

At the energy level, the total potential energy of the system can be written as the sum of magnetic potential and elastic energy:

$$U(\theta) = (1/2)k_1(\theta - \theta_0)^2 - m_1B_1 \cos(\varphi - \theta) \quad \text{(S8)}$$

When $B_1 < B_2$, $U(\theta)$ has only one minimum near θ_0. When $B_1 = B_2$, an inflection point appears adjacent to the original minimum (the critical point). When $B_1 > B_2$, two minima appear (bistable). The transition from monostable to bistable explains the nature of the snap-through from an energy perspective—after crossing the critical point, the system finds a new equilibrium configuration with lower energy, resulting in a rapid configuration transition.

S2. Geometric Stiffness Model of Stepping–Pushing Asymmetry

The corrugated flexural notch structure exhibits significant geometric nonlinearity during bending. When bending toward the notched side (compression side), the notch closes and the material undergoes primarily compressive deformation, resulting in lower equivalent stiffness. When bending away from the notched side (tension side), the notch opens and the material undergoes primarily tensile deformation, resulting in higher equivalent stiffness.

For a single notch segment, the bending stiffness on the compression side and tension side can be approximated as:

$$k_2 = E\, I_1 / L \quad \text{(S9)}$$

$$k_3 = E\, I_2 / L \quad \text{(S10)}$$

where E is the elastic modulus, L is the segment length, and I_1 and I_2 are the cross-sectional second moments of area on the compression side and tension side, respectively. Let the notch depth be d_1 and the wall thickness be t_1. The effective section height on the compression side is $h_1 = t_1 - d_1$, and on the tension side is $h_2 = t_1$. For a rectangular cross-section (width b_1), the second moments of area are:

$$I_1 = (1/12)b_1(t_1 - d_1)^3 \quad \text{(S11)}$$

$$I_2 = (1/12)b_1t_1^3 \quad \text{(S12)}$$

Since notching reduces the effective section height on the compression side, $I_1 < I_2$, and therefore $k_2 < k_3$.

Let the stepping direction (forward bending) correspond to compression-side deformation and the pushing direction (reverse bending) correspond to tension-side deformation. The ratio of equivalent stiffness is:

$$k_3 / k_2 = [t_1/(t_1 - d_1)]^3 = \alpha_1 \quad \text{(S13)}$$

where $\alpha_1 > 1$. Equation (S13) shows that α_1 depends only on the ratio of notch depth to wall thickness d_1/t_1, independent of the elastic modulus—an important theoretical conclusion: the asymmetry is a purely geometric effect, not a material property.

Under the same magnetic torque τ_3, the bending amplitudes satisfy:

$$\Phi_1 = \tau_3 / k_2 \quad \text{(S14)}$$

$$\Phi_2 = \tau_3 / k_3 \quad \text{(S15)}$$

Thus:

$$\Phi_1 / \Phi_2 = k_3 / k_2 = \alpha_1 = [t_1/(t_1 - d_1)]^3 \quad \text{(S16)}$$

From the experimental data, the stepping cumulative deflection angle range is 25°–70° ($\Delta\Phi_1 = 45°$), and the pushing range is 63°–80° ($\Delta\Phi_2 = 17°$), giving:

$$\alpha_1 = \Delta\Phi_1 / \Delta\Phi_2 \approx 45/17 \approx 2.65 \quad \text{(S17)}$$

The geometric parameter relationship can thus be inferred:

$$[t_1/(t_1 - d_1)]^3 = 2.65 \Rightarrow t_1/(t_1 - d_1) \approx 1.384 \Rightarrow d_1/t_1 \approx 0.277 \quad \text{(S18)}$$

This estimated value is in good agreement with the nominal design values (notch depth ≈ 1 mm, wall thickness ≈ 3.6 mm, $d_1/t_1 \approx 0.28$), theoretically demonstrating that "the stepping–pushing asymmetry is determined by the predefined intrinsic curvature, not by material or magnetic field inhomogeneities."

Furthermore, this geometric stiffness model also explains why the "slow-in-the-middle, fast-at-the-ends" angular response curve is directly related to the intrinsic bending angle (115°). Near the intrinsic configuration, the system operates in the lowest stiffness region, and the magnetic torque needs to overcome only a small restoring torque to produce angular changes. Away from the intrinsic configuration, the system enters the rapidly increasing stiffness region, and angular increments require stronger magnetic fields—this stiffness modulation is a direct manifestation of the geometric nonlinearity of the corrugated notches.

S3. Hydrodynamic Analysis of Swimming Propulsion

Under fully submerged conditions, the primary resistance to the robot's forward motion at speed v_1 is hydrodynamic drag:

$$F_1 = (1/2)\rho_1 C_1 A_1 v_1^2 \quad \text{(S19)}$$

where $\rho_1 \approx 1000$ kg/m³ is the density of water, A_1 is the frontal area of the robot (≈ 25 mm × 18 mm ≈ 4.5×10^{-4} m²), and C_1 is the drag coefficient. At Reynolds numbers $Re_1 = \rho_1 v_1 L_1/\mu_1$ ($L_1 \approx 0.04$ m, $\nu_1 = 1\times10^{-6}$ m²/s, $v_1 \approx 0.02$–0.06 m/s, $Re_1 \approx 800$–2400), for a flattened shape, $C_1 \approx 1.0$–1.2.

The propulsive force originates from the momentum change generated by the leg paddling motion. Let the effective paddling area of a single leg be A_2 and the paddling speed be u_1 (determined by the magnetic field frequency f_1 and the swing amplitude $\Delta\Phi_3$, $u_1 \approx f_1 \cdot \Delta\Phi_3 \cdot L_2$). The propulsive force can be approximated as:

$$F_2 \approx (1/2)\rho_1 C_2 A_2 u_1^2 \quad \text{(S20)}$$

At steady swimming, $F_2 = F_1$:

$$(1/2)\rho_1 C_2 A_2 u_1^2 = (1/2)\rho_1 C_1 A_1 v_1^2 \quad \text{(S21)}$$

This simplifies to:

$$v_1 \approx u_1 \cdot \mathrm{sqrt}(\ (C_2 A_2) / (C_1 A_1)\ ) \quad \text{(S22)}$$

From the experimental data: the rear leg cumulative deflection angle amplitude is 60° (≈ 1.05 rad), leg length $L_2 \approx 18$ mm, frequency $f_1 = 0.8$ Hz, paddling speed $u_1 \approx f_1 \cdot \Delta\Phi_3 \cdot L_2 \approx 0.8 \times 1.05 \times 0.018 \approx 0.015$ m/s. Substituting $A_1 \approx 4.5\times10^{-4}$ m², $A_2 \approx$ leg width × stroke ≈ 5 mm × 18 mm ≈ 9×10^{-5} m², and taking $C_2 \approx C_1 \approx 1.0$ gives:

$$v_1 \approx 0.015 \times \mathrm{sqrt}(9\times10^{-5} / 4.5\times10^{-4}) \approx 0.015 \times 0.447 \approx 0.0067 \text{ m/s} \approx 6.7 \text{ mm/s} \quad \text{(S23)}$$

This estimate (≈ 7 mm/s) is of the same order of magnitude as the experimentally measured speed of approximately 12 mm/s at 0.8 Hz and 180 mT. The discrepancy may arise from multi-leg cooperative propulsion (four legs paddling simultaneously rather than a single leg), unsteady effects (added mass effects during paddling can increase effective propulsion), and dynamic changes in effective paddling area during the stroke.

It is worth emphasizing that at 340 mT and 0.8 Hz, the speed reaches 56 mm/s—approximately eight times the estimated value. The primary reason is the simultaneous increase in paddling amplitude and speed with magnetic field intensity: as the field intensity increases from 180 mT to 340 mT, the rear leg cumulative deflection angle amplitude increases from approximately 60° to the experimental maximum, and the paddling speed u_1 also increases. Additionally, at higher field strengths, the leg motion approaches "rapid flapping" rather than "slow swinging," and unsteady fluid effects (such as vortex ring formation) can further enhance propulsion. This multi-mechanism synergy explains the nonlinear increase in swimming speed with magnetic field intensity observed experimentally.

From a fluid dynamics perspective, the high propulsive efficiency of the paddling mode can also be explained by the Strouhal number:

$$St_1 = f_1 A_3 / v_1 \quad \text{(S24)}$$

where f_1 is the paddling frequency and A_3 is the wake width (≈ leg swing amplitude). For efficient biological propulsion, St_1 typically falls in the range of 0.2–0.4. Substituting $f_1 \approx 0.8$ Hz, $A_3 \approx 18$ mm × sin(60°) ≈ 15.6 mm, and $v_1 \approx 56$ mm/s gives $St_1 \approx 0.8 \times 0.0156 / 0.056 \approx 0.22$, which falls exactly within the efficient propulsion range, further validating the hydrodynamic rationality of this paddling mode.

S4. Torque Balance Analysis of Single-Sided Self-Righting

After the robot overturns, the flexible torso contacts the ground and the four legs are in an irregularly splayed state. Let the robot width be w_1 (25 mm), length be l_1 (40 mm), and mass be m_2 (from the volume $40\times25\times18\ mm^3 \approx 18\ cm^3$ and density $\rho_2 \approx 1.15\ g/cm^3$, $m_2 \approx 20.7$ g). In the overturned posture, the robot contacts the ground along one long edge, and the horizontal distance from the center of gravity to this pivot point, d_2, depends on the torso torsion and the offset of the center of gravity. Conservatively, $d_2 \approx w_1/4 \approx 6.25$ mm.

The gravitational torque is:

$$\tau_4 = m_2 g_1 d_2 \approx 0.0207 \times 9.8 \times 0.00625 \approx 1.27 \times 10^{-3}\ N\cdot m \qquad (S25)$$

The two legs on one side bend under the magnetic field, generating a magnetic driving force F_3 acting on the underside of that side of the torso. The restoring torque is:

$$\tau_5 = 2F_3 \cdot (w_1/2) = F_3 w_1 \qquad (S26)$$

The factor of 2 accounts for the cooperative contribution of the two legs. The righting condition is:

$$\tau_5 > \tau_4 \qquad (S27)$$

Substituting $w_1 = 0.025$ m:

$$F_3 > \tau_4 / w_1 \approx 1.27\times10^{-3} / 0.025 \approx 0.051\ N \qquad (S28)$$

That is, each leg on the single side needs to provide approximately 0.025 N of magnetic driving force. Under a 300 mT magnetic field, the magnetic driving force from the magnetized particle chains can reach tens of mN, making this condition feasible.

Further considering the dynamics of the righting process, let the moment of inertia of the robot about the pivot point be I_3 (for an approximately rectangular plate rotating about an edge, $I_3 \approx (1/3)m_2 l_1^2 \approx 0.333 \times 0.0207 \times 0.04^2 \approx 1.10\times10^{-5}\ kg\cdot m^2$). The righting angular acceleration α_2 is given by the torque equation:

$$\alpha_2 = (\tau_5 - \tau_4) / I_3 \qquad (S29)$$

Near the completion of flipping, the gravitational torque gradually decreases to zero while the net torque increases, so the righting process exhibits accelerating behavior. This analysis explains the experimentally observed time scale of "overturning at 2 s → righting at 8 s"—a flipping time of approximately 6 s is consistent with the time constant (on the order of 1–10 s) determined by mN-scale torque and $10^{-5}\ kg\cdot m^2$-scale moment of inertia.

If both sides were actuated simultaneously, the restoring torques from the two sides would be opposite in direction and equal in magnitude, resulting in zero net torque:

$$\tau_6 = \tau_{5,left} - \tau_{5,right} \approx 0 \qquad (S30)$$

The torso would experience no net flipping torque and would be pulled in opposite directions by the legs on both sides, leading to a locked state. Only single-sided actuation breaks the torque balance and generates a net flipping torque. This theoretical derivation is

fully consistent with the experimental observations, demonstrating from fundamental mechanical principles the necessity of "single-sided rather than double-sided actuation."

Furthermore, the effectiveness of the single-sided actuation strategy also benefits from the torsional deformation capability of the flexible torso. Under the action of a single-sided bending moment, the flexible torso can undergo local torsion, shifting the center of gravity further outward from the pivot point and reducing the required torque threshold for flipping. Greater flexibility leads to larger d_2 and smaller gravitational torque $\tau_4 = m_2 g_1 d_2$, making righting easier. This reveals another important function of the flexible torso in this design—it is not merely a structural connector but also a passive participant in the self-righting mechanism.

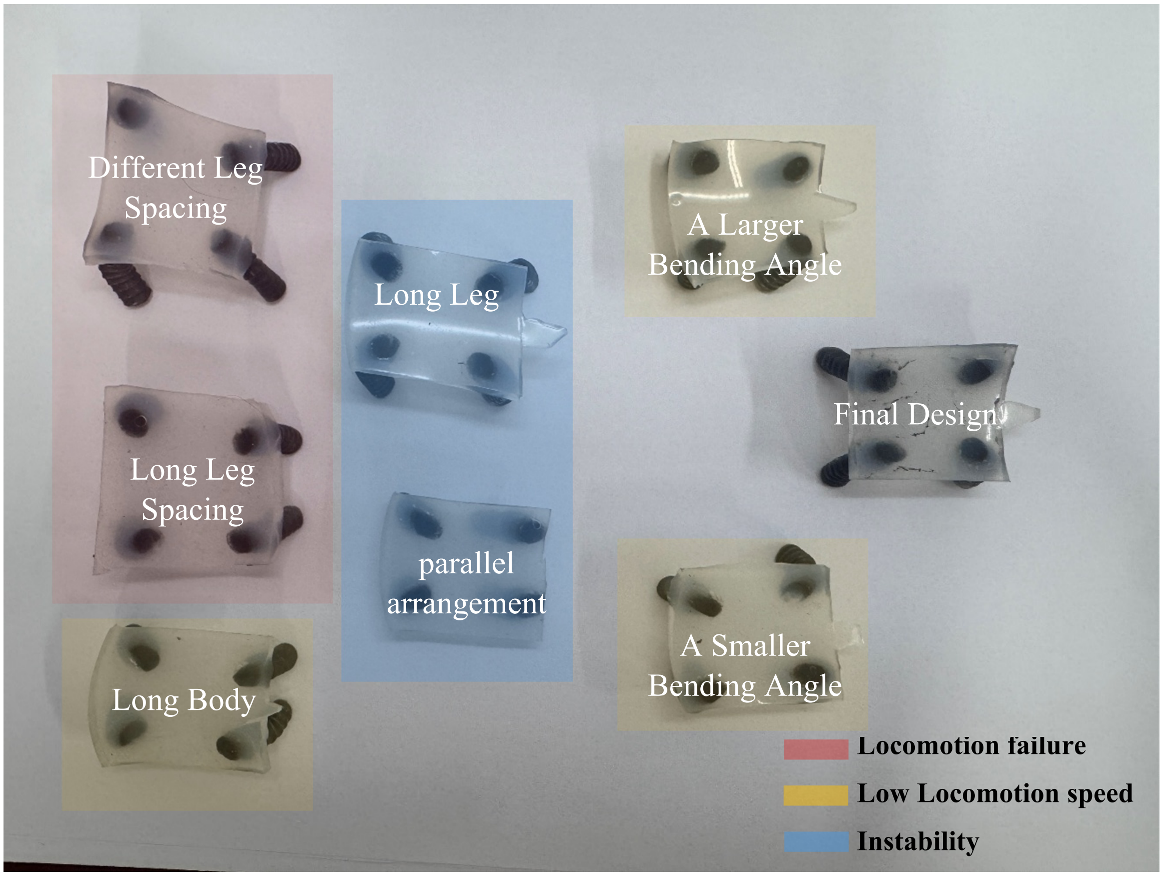

Supplementary Figure S1. Fabricated soft robots with different structural designs. Representative prototypes with different leg spacing, leg length, body length, leg arrangement, and intrinsic bending angle were fabricated to investigate the effects of structural parameters on locomotion performance and to determine the final robot configuration.

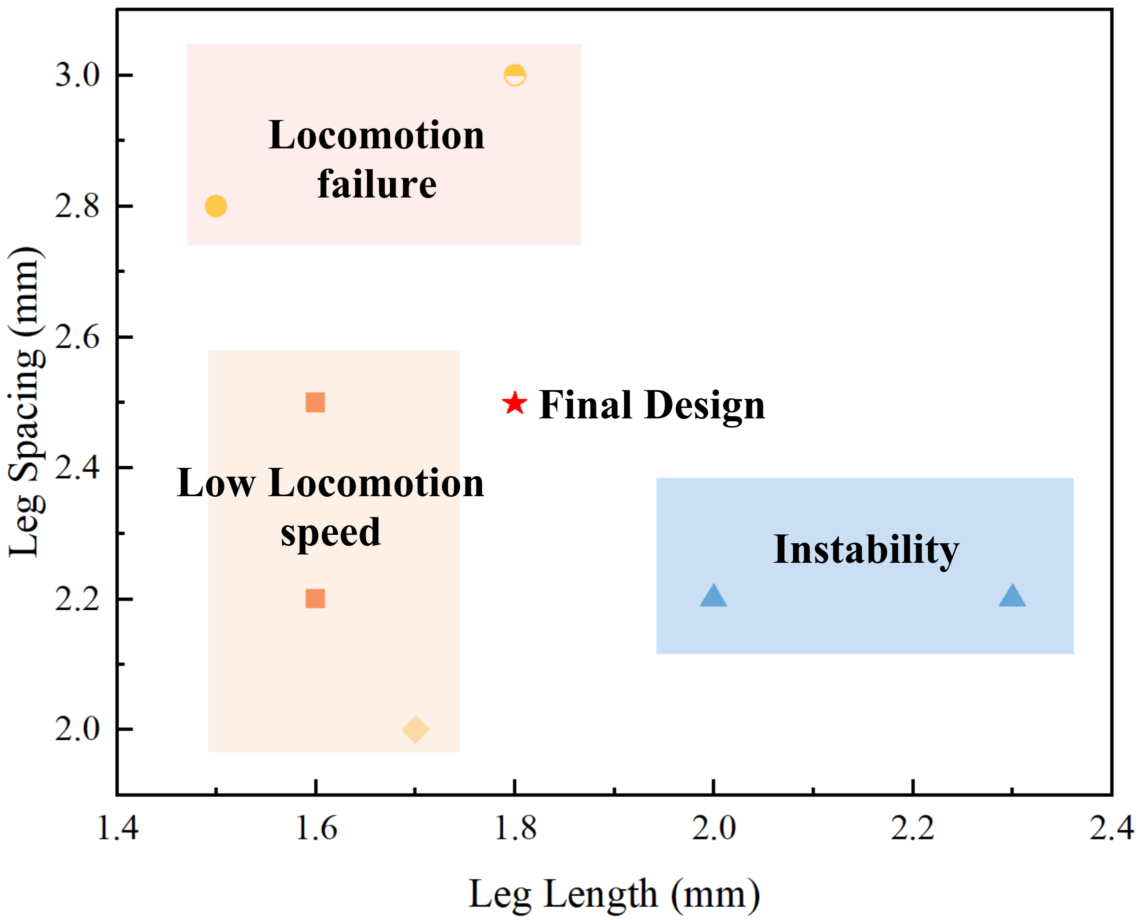

Supplementary Figure S2. Design-space screening based on leg length and leg spacing. Different combinations of leg length and leg spacing were evaluated according to locomotion performance and structural stability. The tested designs can be categorized into locomotion failure, low locomotion speed, and instability regions. The final design, with a 1.8 mm leg length and 2.5 mm leg spacing, is located within the suitable design region and provides a balanced configuration for stable locomotion.

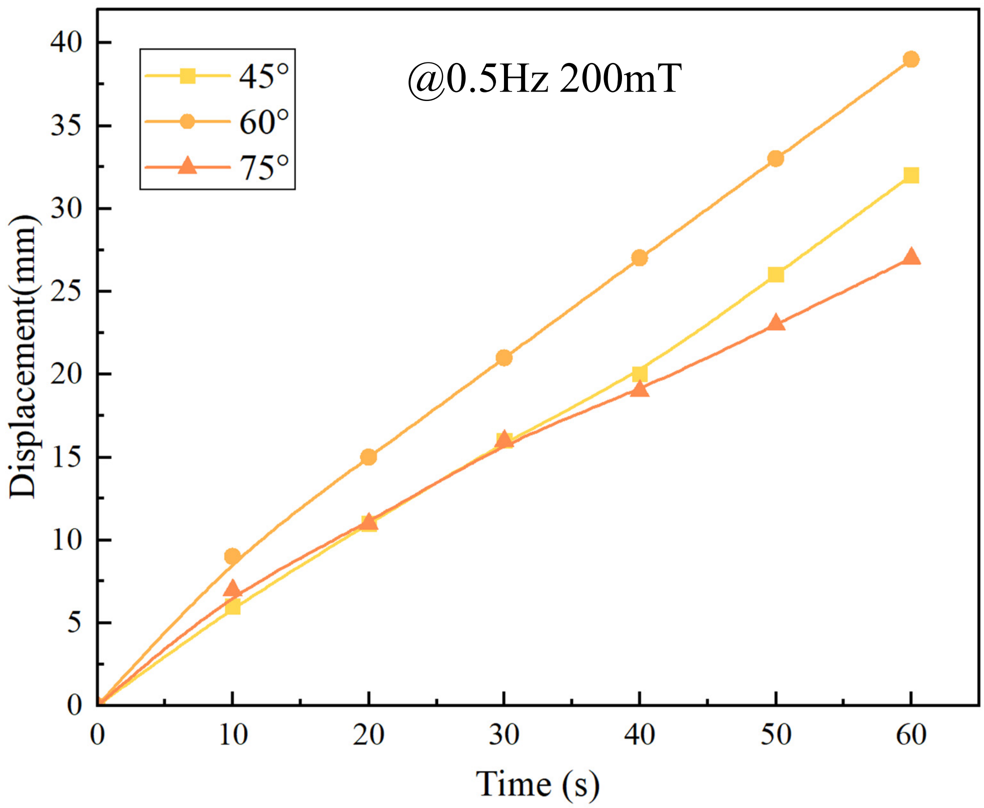

Supplementary Figure S3. Effect of intrinsic bending angle on locomotion performance. Displacement of robots with different intrinsic bending angles (45°, 60°, and 75°) under a magnetic field of 200 mT at 0.5 Hz. The 60° configuration produces the largest displacement and is therefore selected as the optimal intrinsic bending angle for the final design.
